# Draft Genome Assembly and Annotation of a Male Mezayen Dromedary Camel of the Majaheem Type (*Camelus dromedarius*)

**DOI:** 10.3390/ani16182905

**Published:** 2026-09-15

**Authors:** Fahad H. Alqahtani, Ahmad M. Aldossary, Fahad M. Alhoshani, Ion I. Măndoiu, Mohamed B. Al-Fageeh, Manee M. Manee, Badr M. Al-Shomrani

**Affiliations:** 1 National Center for Bioinformatics, King Abdulaziz City for Science and Technology, Riyadh 11442, Saudi Arabia; fqahtani@kacst.gov.sa; 2 Advanced Agricultural and Food Technologies Institute, King Abdulaziz City for Science and Technology, Riyadh 11442, Saudi Arabia; 3 Wellness and Preventative Medicine Institute, King Abdulaziz City for Science and Technology, Riyadh 11442, Saudi Arabia; 4 Advanced Diagnostic and Therapeutic Technologies Institute, King Abdulaziz City for Science and Technology, Riyadh 11442, Saudi Arabia; fhoshani@kacst.gov.sa; 5 Department of Computer Science and Engineering, University of Connecticut, 371 Fairfield Way, Unit 4155, Storrs, CT 06269, USA; ion@engr.uconn.edu

**Keywords:** Arabian camel, dromedary, Mezayen, Majaheem type, genome assembly, selective breeding

## Abstract

The Arabian camel (*Camelus dromedarius*), notably the show camels of the Mezayen tradition, holds significant cultural and socio-economic importance in the Middle East. Mezayen is a camel beauty contest tradition highlighting the remarkable aesthetic qualities of these animals. Despite the importance of these show camels, their genetic characteristics have been minimally explored. Currently, a reference genome assembly for Mezayen Arabian camels is lacking, creating a critical gap in understanding camelid genetics. This gap poses a significant barrier to advancements in selective breeding and conservation of this culturally and economically valuable species. To bridge this gap, we sequenced, assembled, and annotated the first draft scaffold-level genome assembly of a male Mezayen camel (Majaheem type). Employing a multi-platform genomic assembly strategy, we combined PacBio long-read sequencing, Illumina short-read polishing, and 10x Genomics linked-read scaffolding to achieve a high-quality draft scaffold-level genome assembly for the Mezayen camel. BUSCO analysis confirmed that the genome assembly contains 97.7% of the expected complete orthologous genes (single-copy and duplicated), indicating a high level of genomic completeness. The final genome assembly is 2.01 Gb in size, with a scaffold N50 of 53.40 Mb, and comprises 24,899 non-redundant protein-coding genes. This assembly achieves the highest contig N50 among the five dromedary assemblies evaluated in this study and ties for the highest overall BUSCO completeness, significantly enhancing the genomic resources available for this vital animal. The findings of this study provide a foundational genomic resource that can support future conservation and marker-assisted selection efforts for these culturally esteemed camels. We note that the annotation was generated without RNA-seq evidence; incorporating transcriptomic data is an important direction for refining gene models in future work.

## Simple Summary

This study explores the genomics of Mezayen Arabian camels, which have important cultural and economic value in the Middle East. Despite their importance, genomic information representing these camels remains limited. To address this gap, we generated and characterized the first draft scaffold-level genome assembly of a male Mezayen camel of the Majaheem type using complementary sequencing technologies. The resulting high-quality genome assembly and gene annotations provide an important genomic resource for studying genetic diversity and the inheritance of economically and culturally important traits. This resource can support future studies on parentage, genetic diversity, conservation, and selective breeding and provides a foundation for broader population-level genomic research in Mezayen and Arabian camels.

## 1. Introduction

The family Camelidae encompasses a phylogenetically diverse group of large ungulates distributed across arid and semi-arid regions of Asia, North Africa, South America, and Australia [1]. Extant members of the family Camelidae belong to two tribes, Camelini (Old World camels) and Lamini (New World camelids), which were estimated to have diverged approximately 15.8 million years ago (Mya) based on mitochondrial sequence analysis [2]. The Camelini tribe comprises three species, namely the dromedary or Arabian camel (*Camelus dromedarius*), the two-humped Bactrian camel (*Camelus bactrianus*), and the wild Bactrian camel (*Camelus ferus*), while the Lamini tribe includes two wild species, the vicuña (*Vicugna vicugna*) and the guanaco (*Lama guanicoe*), alongside two domesticated species, the llama (*Lama glama*) and the alpaca (*Vicugna pacos*). Across these lineages, camelids have evolved a suite of remarkable physiological and biochemical adaptations that confer resilience to extreme desert conditions, including prolonged food and water deprivation, wide thermal fluctuations, and exposure to high solar irradiance [3,4].

Among camelid species, the dromedary (*C. dromedarius*) is numerically and economically dominant, accounting for approximately 53% of Saudi Arabia’s total camel population as reported by [5] and occupying arid zones throughout Asia and North Africa [5]. Within this species, camels of the Mezayen show tradition occupy a particularly prominent cultural and socio-economic position in the Arabian Peninsula and broader Gulf region. Mezayen show camels are selectively bred for highly specific aesthetic and morphological standards, including coat color, ear length, neck curvature, and overall body proportion (aesthetic standards judged in competition rather than quantitatively defined production traits), and compete in formal beauty contests that have evolved into a major regional industry spanning Saudi Arabia, Kuwait, and the United Arab Emirates (UAE) [6]. These show camels are classified into recognized types (coat-color-defined categories within the show-camel group rather than genetically distinct breeds, populations, or ecotypes) based on these phenotypic criteria, of which the Majaheem, Waddah, and Awadi are among the most prominent [7,8]. These types are broadly grouped by coat color into the black-coated Majaheem and the lighter, colored Malaween types (such as Waddah), a distinction referred to later in this study. Despite this cultural prominence and the considerable economic stakes associated with Mezayen competition, the genetic and genomic basis of their defining phenotypic traits remains poorly characterized. A recent mitochondrial study examining haplotype diversity among Mezayen camel types revealed significant intra-type genetic variability and identified two major haplogroups, while also highlighting unexpected genetic homogeneity across types of differing coat color from the Arabian Peninsula [9]. However, mitochondrial analyses alone are insufficient to resolve the nuclear genomic architecture underlying complex morphological traits, and no nuclear reference genome from a Mezayen individual existed prior to the present study.

The broader genomic landscape of *C. dromedarius* has advanced considerably over the past decade, driven by improvements in sequencing technology and assembly methodology. The first published dromedary genome assembly, generated by Wu et al. [4] from short-read Illumina data, provided a foundational resource but achieved a BUSCO completeness of only 90.9% and a contig N50 of 0.07 Mb, reflecting the well-documented contiguity limitations of short-read-only strategies. A subsequent assembly of the North African dromedary (CamDro2, later updated to CamDro3) integrated Illumina sequencing with Hi-C and long-read PacBio data, substantially improving assembly quality to a BUSCO score of 97.7% and achieving chromosome-level scaffolding [10]. More recently, two haplotype-resolved chromosome-level assemblies of a male dromedary were deposited in the NCBI under the Vertebrate Genomes Project (mCamDro1.hap2, GCA_036321565.1; mCamDro1.pat, GCA_036321535.1), representing the current state of the art for dromedary genome resources. Together, these assemblies have substantially advanced understanding of camelid evolution, arid-environment adaptation, and comparative genomics [4,10]. Nevertheless, because all existing reference genomes derive from non-Mezayen individuals (primarily of African or non-pedigreed Arabian origin), genomic resources for this culturally and economically significant group of camels are lacking.

This gap has practical consequences. The absence of a reference genome from a Mezayen individual has limited genome-wide association studies aimed at resolving the genetic architecture of Majaheem competition traits, the application of marker-assisted selection in breeding programs, and population-level resequencing analyses. The reference framework presented here is a necessary prerequisite for such studies, but is not on its own sufficient to identify trait-associated variants, which will require population-level resequencing with adequate sample sizes and reliable phenotype records. Although genome-wide selection signature analyses in *C. dromedarius* have identified candidate loci associated with coat color pigmentation (including variants in *MC1R* and *ASIP*), immune function, and metabolic adaptation in African and Arabian Peninsula populations [11,12,13], these findings have not been extended to Majaheem-type camels. Such analyses require a conspecific reference assembly as a prerequisite, which the present study provides. Investigation of these candidate loci in Majaheem-type camels is therefore a direction for future work rather than an aim of the present study.

To address this critical gap, we present the first draft scaffold-level genome assembly of a male Mezayen dromedary of the Majaheem type, the primary participant in formal beauty contests, designated Mezayen_CamDro_v1. Employing an integrative multi-platform strategy that combines PacBio long-read sequencing, Illumina short-read polishing, and 10x Genomics linked-read scaffolding, we generated a high-quality scaffold-level assembly with a total length of 2.01 Gb, a contig N50 of 40.04 Mb, and a BUSCO completeness score of 97.7%. The assembly was annotated using a hybrid pipeline integrating homology-based gene transfer and ab initio prediction via BRAKER3, producing a final consensus set of 24,899 non-redundant protein-coding genes with high functional coverage. Repetitive element content and simple sequence repeat (SSR) profiles were further characterized to provide a comprehensive genomic resource applicable to population genetics, breed traceability, and marker-assisted selection. The Mezayen_CamDro_v1 assembly achieves the highest contig N50 among all five dromedary assemblies evaluated in this study and ties for the highest overall BUSCO completeness, establishing it as a foundational genomic resource for advancing selective breeding, conservation, and trait investigation in these culturally esteemed camels.

## 2. Materials and Methods

### 2.1. Sample Collection and DNA Isolation

Blood was collected from Hakaman, a male Mezayen show camel of the Majaheem type, bred at Mohamed bin Abdullah Al-Fassam’s camel breeding farm in Wadi Aldwaser, Saudi Arabia, which secured second place in the Majaheem category at the King Abdulaziz Camel Festival in December 2019. The animal was farm-bred and maintained under standard husbandry conditions typical of camel breeding operations in the region, with routine access to feed and water. The exact age of the animal was not recorded and is therefore not available; the camel was an adult male in good general health at the time of sampling, with no known illness or prior experimental procedures.

Blood was collected by trained personnel during routine handling, with the animal manually restrained using standard handling techniques and a single sample drawn from the jugular vein into anticoagulant-treated tubes using aseptic technique. No sedation was required, and the sampling caused only minimal, transient discomfort. High-molecular-weight (HMW) DNA was isolated in four independent extractions from the same individual camel using the MagAttract HMW DNA Kit (Qiagen, Hilden, Germany) according to the manufacturer’s protocol, and the extractions were submitted together for sequencing. DNA concentration was quantified using a Qubit fluorometer, and purity was assessed from NanoDrop absorbance ratios. Across the four extractions, Qubit concentrations ranged from 40.9 to 72.9 ng/µL, A260/280 ratios from 1.77 to 1.81, and A260/230 ratios from 2.04 to 2.30. DNA integrity was assessed by gel electrophoresis and was consistent with high-molecular-weight DNA, further supported by the length of the resulting PacBio reads (subread N50 13.4 kb; Supplementary Table S3).

### 2.2. Genome Sequencing

Genomic sequencing was conducted at the Georgia Genomics and Bioinformatics Core (GGBC), University of Georgia, USA, using three complementary platforms: Illumina, PacBio, and 10x Genomics, targeting ∼25×, ∼60–80×, and ∼50× genome coverage, respectively, estimated against an expected genome size of approximately 3 Gb. For Illumina sequencing, a paired-end library (2×150 bp) was prepared according to the manufacturer’s protocol for the Kapa Hyper Prep Kit (Kapa Biosystems, Wilmington, MA, USA) and sequenced on the Illumina HiSeq 3000/4000. For PacBio long-read sequencing, fragments smaller than 20 kb were removed from purified genomic DNA using the BluePippin instrument (Sage Science, Beverly, MA, USA). The size-selected DNA was then used to prepare a library with the SMRTbell Express Template Prep Kit 2.0, which was loaded onto a Sequel SMRT Cell 1M v2 and sequenced using the Sequel Sequencing Kit 2.0. For 10x Genomics sequencing, Gel Bead-In-Emulsions (GEMs) were generated using the Chromium Genome Chip, and libraries were constructed following the Chromium Genome Reagent Kit Protocol v2. Sequencing was performed on the Illumina NextSeq 500 with 2×150 bp paired-end reads.

### 2.3. Genome Assembly, Scaffolding, and Quality Assessment

The genome assembly of the Mezayen camel was achieved through an integrative multi-platform approach. The initial draft assembly was generated from the pacbio_1 long-read PacBio CLR dataset using nextDenovo v2.5.2 (https://github.com/Nextomics/NextDenovo (accessed on 10 February 2026)), a tool designed specifically for long-read data that adopts a “correct-then-assemble” strategy, offering improved efficiency and reduced computational requirements compared to alternative assemblers such as Canu [14]. NextDenovo was configured for CLR reads with a genome-size parameter of 3 Gb and a read-length cutoff of 1 kb; applying this cutoff retained 226.12 Gb of PacBio data, corresponding to approximately 75.4× theoretical input coverage relative to the 3 Gb parameter. Following draft generation, a polishing step was performed using Pilon v1.24 [15], in which short-read Illumina data were aligned against the draft assembly to correct residual sequencing errors, including mismatches, indels, and block substitutions. For this polishing step, Illumina reads were aligned with HISAT2 v2.2.1 [16] (options –no-discordant, –no-mixed, –sensitive, -p 64, –mm); the paired-end dataset comprised 253,656,416 151-bp reads (38.30 Gb), yielding 222,024,848 primary mapped reads (33.12 Gb CIGAR-mapped; approximately 16.49× effective polishing depth). The effect of polishing was evaluated post hoc, without modifying the final assembly, by comparing the pre- and post-Pilon assemblies with QUAST v5.2.0 [17] and assessing consensus quality with Merqury on a common Illumina-derived Meryl k-mer database (k=21). The final assembly phase involved scaffolding the polished contigs with 10x Genomics linked-read data using Scaff10X v5.0 (https://github.com/wtsi-hpag/Scaff10X (accessed on 10 February 2026)) with internal parameters -file 1, -matrix 2000, -link 8, -uplink 50, and -longread 1, which reduces fragmentation by organizing and joining contigs into larger, more contiguous scaffolds. This multi-step pipeline, integrating long-read assembly, short-read polishing, and linked-read scaffolding, leveraged the complementary strengths of each technology to produce a high-quality draft genome of the Mezayen camel.

Assembly completeness and gene content were evaluated using the Benchmarking Universal Single-Copy Orthologs (BUSCO) framework v5.8.0 [18], run locally in genome mode (euk_genome_min) with miniprot as the gene predictor. We selected the *Artiodactyla* lineage dataset from OrthoDB v12 [19], comprising 12,594 conserved single-copy orthologous genes (dataset creation date: 1 July 2025), as it represents the most current and taxonomically specific lineage available for dromedary camel genome assessment. This replaces the previously used Cetartiodactyla ortholog set (OrthoDB v10; 13,335 genes), providing a more refined and up-to-date benchmark for camelid genomes. BUSCO scores were calculated for the Mezayen_CamDro_v1 assembly and compared against four publicly available dromedary camel genome assemblies: the North African Dromedary (CamDro3; GCA_000803125.3) [10], the Arabian Dromedary (Ca_dromedarius_V1; GCA_000767585.1) [4], and two haplotype-resolved assemblies from the Vertebrate Genomes Project, mCamDro1.hap2 (GCA_036321565.1) and mCamDro1.pat (GCA_036321535.1). All five assemblies were downloaded from NCBI on 5 April 2026 using the datasets command-line tool v14 and processed under identical BUSCO parameters to ensure comparability. Each assembly was provided as its unmasked genomic FASTA without deduplication or filtering, and BUSCO v5.8.0 was run in genome mode (euk_genome_min) against artiodactyla_odb12 using miniprot v0.18-r281 as the gene predictor with 8 threads. This comparative analysis benchmarks the Mezayen_CamDro_v1 assembly against the current state of dromedary camel genomic resources, providing a standardized assessment of gene content completeness and assembly quality.

Assembly quality was further evaluated using complementary reference-free and read-based approaches. A k-mer database was built from the Illumina reads using Meryl v1.3 (k=21), and consensus quality value (QV) and k-mer completeness were estimated with Merqury v1.3 [20]. Heterozygosity and haploid genome size were modelled from the same k-mer histogram using GenomeScope2 v2.1.0 [21] (ploidy = 2). Illumina reads were mapped to the final assembly with BWA-MEM2 v2.2.1 [22], and PacBio reads with minimap2 v2.31 [23] (map-pb preset); mapping statistics and per-scaffold depth were summarized using SAMtools and mosdepth v0.3.13 [24]. Residual haplotypic duplication was assessed with purge_dups v1.2.6 [25], and candidate duplicated scaffolds were validated independently using PacBio read depth and pairwise sequence alignment.

Contamination and redundancy were assessed at the scaffold level. Taxonomic screening was performed with Kraken2 v2.17.1 [26] (PlusPF database) on both the final assembly and the raw Illumina and PacBio reads, at confidence thresholds of 0.05 and 0.10. Scaffold GC content, coverage, and Kraken2 classifications were integrated with BlobTools2 v4.5.1 [27]. An independent mitochondrial genome was assembled from the same individual with NOVOPlasty [28] and compared with the nuclear assembly using BLASTN [29] to distinguish redundant mitochondrial sequences from nuclear mitochondrial insertions (NUMTs), with candidate loci validated using spanning PacBio reads. Vector and adaptor contamination were screened with NCBI FCS-adaptor v0.5.5 in eukaryotic mode. Residual haplotypic duplication was evaluated with purge_dups v1.2.6 [25] as described above.

To further evaluate the structural integrity and contiguity of the Mezayen_CamDro_v1 assembly, whole-genome synteny was assessed against the chromosome-level paternal haplotype assembly mCamDro1.pat (GCA_036321535.1), which served as a high-quality reference. Pairwise sequence alignments were generated using minimap2 v2.30 [23] with the assembly-to-assembly preset (-x asm5) and secondary alignments suppressed (–secondary=no). Alignments were filtered to a minimum length of 10 kb and a mapping quality of ≥30. The 28 largest Mezayen_CamDro_v1 scaffolds (≥20 Mb), representing the major chromosome-scale sequences of the assembly, were selected for synteny visualization. Synteny ribbon plots were generated in R v4.5.2 using ggplot2 [30] and patchwork, with Bezier curves connecting syntenic alignment blocks between the Mezayen scaffolds and the 38 chromosomes of mCamDro1.pat.

### 2.4. Genome Annotation

Repetitive elements were identified and masked prior to gene prediction. De novo repeat families were modeled using RepeatModeler v2.0.7 [31] with LTR structural detection enabled. The resulting custom repeat library was used by RepeatMasker v4.2.3 [32] (-xsmall -gff -no_is -e ncbi) to produce a soft-masked genome; lowercase soft-masked A/C/G/T bases represented 26.30% of the assembly, preserving repeat sequence in lowercase for repeat-aware gene prediction.

Gene prediction followed a hybrid strategy combining homology-based gene transfer and protein-supported ab initio prediction. Protein-coding gene models were transferred from the NCBI RefSeq-annotated dromedary reference genome (mCamDro1.pat; GCF_036321535.1) using Liftoff v1.6.3 [33] with the -exclude_partial flag, and the resulting GFF3 was normalized with AGAT v0.8.1 [34]. Independent ab initio prediction was performed with BRAKER v3.0.8 [35] on the soft-masked genome, using the OrthoDB v11 Vertebrata protein set [19] as the sole extrinsic evidence and the species model camelus_dromedarius_mezayen, with 48 threads. No RNA-seq evidence or BUSCO hints were supplied. BRAKER was run in protein-only EP mode, comprising GeneMark-ES, ProtHint protein-hint generation, and GeneMark-EP, followed by AUGUSTUS [36] training and prediction and TSEBRA reconciliation.

The Liftoff, AUGUSTUS, and GeneMark predictions were integrated into a consensus gene set using EvidenceModeler v2.1.0 (EVM) [37] with relative weights of 5:2:2 (Liftoff:AUGUSTUS:GeneMark), a 5 Mb segment size, 500 kb overlaps, a minimum intron length of 20 bp, and a terminal intergenic re-search of 10 kb; no user-defined score, length, or deduplication cutoff was applied prior to integration. To assess sensitivity to the weighting scheme, EVM was additionally run with weights of 3:2:2 and 1:1:1 under otherwise identical parameters.

Functional annotation of the predicted proteome was performed using two complementary approaches. Predicted protein sequences were searched against the UniProtKB/Swiss-Prot database using DIAMOND BLASTp v2.0.14 [38] with an e-value threshold of $1\times 10^{-5}$, retaining the top hit per query. eggNOG-mapper v2.1.12 [39] was run against the eggNOG v5.0.2 database [40] in DIAMOND mode (--tax_scope Mammalia; --go_evidence all), providing Gene Ontology (GO) [41], KEGG [42], and Pfam [43] annotations.

Annotation completeness was assessed using BUSCO v5.8.0 [18] in protein mode against the *Artiodactyla* lineage dataset (artiodactyla_odb12; OrthoDB v12.1; 12,594 orthologs). Proteome quality and taxonomic consistency were independently evaluated using OMArk v0.4.1 [44] with OMAmer v2.1.2 against the OMA LUCA database.

### 2.5. Simple Sequence Repeat Identification

Simple sequence repeats (SSRs) were identified in the soft-masked Mezayen_CamDro_v1 genome assembly using the MIcroSAtellite identification tool (MISA) v2.1 [45]. The minimum number of repeat units required for SSR identification was set as follows: 10 repeats for mononucleotide motifs, 6 repeats for dinucleotide motifs, and 5 repeats for trinucleotide, tetranucleotide, pentanucleotide, and hexanucleotide motifs. Compound SSRs, defined as two or more SSRs interrupted by no more than 100 bp, were also identified and reported separately. SSR abundance, class distribution, and motif frequencies were summarized from the MISA statistics output. SSRs were catalogued as detected by MISA without additional post-hoc filtering: loci were not separately screened against assembly gaps or transposable elements, were not scored for genomic uniqueness, and were not partitioned by genic context. Validation of these candidate loci as polymorphic markers is left to future work. All visualizations of SSR content and genomic repeat composition were produced in R v4.3.3 [46].

## 3. Results

### 3.1. Assembly and Quality Evaluation of the Draft Genome

A whole-genome shotgun sequencing strategy was employed to generate genomic data for the Mezayen dromedary camel (Mezayen_CamDro_v1). The genome assembly was constructed using a hybrid approach integrating datasets from three complementary next-generation sequencing platforms: Illumina, 10x Genomics, and PacBio. Illumina sequencing yielded 151.58 Gb of high-accuracy paired-end short-read data (151-bp reads), 10x Genomics produced 111.25 Gb of linked-read data, and PacBio sequencing generated 384.54 Gb of long-read data across two CLR datasets. The initial de novo assembly used the pacbio_1 dataset (22,414,076 reads; 226.46 Gb). This multi-platform strategy combined the precision of short reads for error correction with the structural resolution of long reads, resulting in a high-quality draft scaffold-level genome assembly with a total sequence length of 2.01 Gb, a contig N50 of 40.04 Mb, and a scaffold N50 of 53.40 Mb (Table 1). Notably, the Mezayen_CamDro_v1 assembly achieved the highest contig N50 among all five dromedary assemblies evaluated in this study, reflecting the superior contiguity afforded by the PacBio long-read sequencing strategy. The contribution of the 10x Genomics scaffolding step was quantified from the retained AGP: 323 pre-scaffolding contigs were incorporated into 278 scaffold objects through 45 joins, each introducing a fixed 100-bp gap (4500 Ns in total, accounting exactly for the difference between the pre-scaffolding and final assembly lengths). Of the 323 input contigs, 248 (76.78%) remained as single-contig scaffolds and 75 (23.22%) were joined into 30 multi-contig scaffolds. Independent PacBio validation, requiring long reads to span an introduced gap with at least 1 kb aligned on each side, supported 39 of the 45 joins (86.7%); the remaining joins were not independently validated by continuous long-read spanning and are reported as such. Across the 278 scaffolds, the median scaffold length was 440.1 kb, and the smallest was 14.3 kb (mean 7.22 Mb), and the 28 largest scaffolds (≥20 Mb) accounted for 75.8% of the total assembly length.

**Figure 1:**
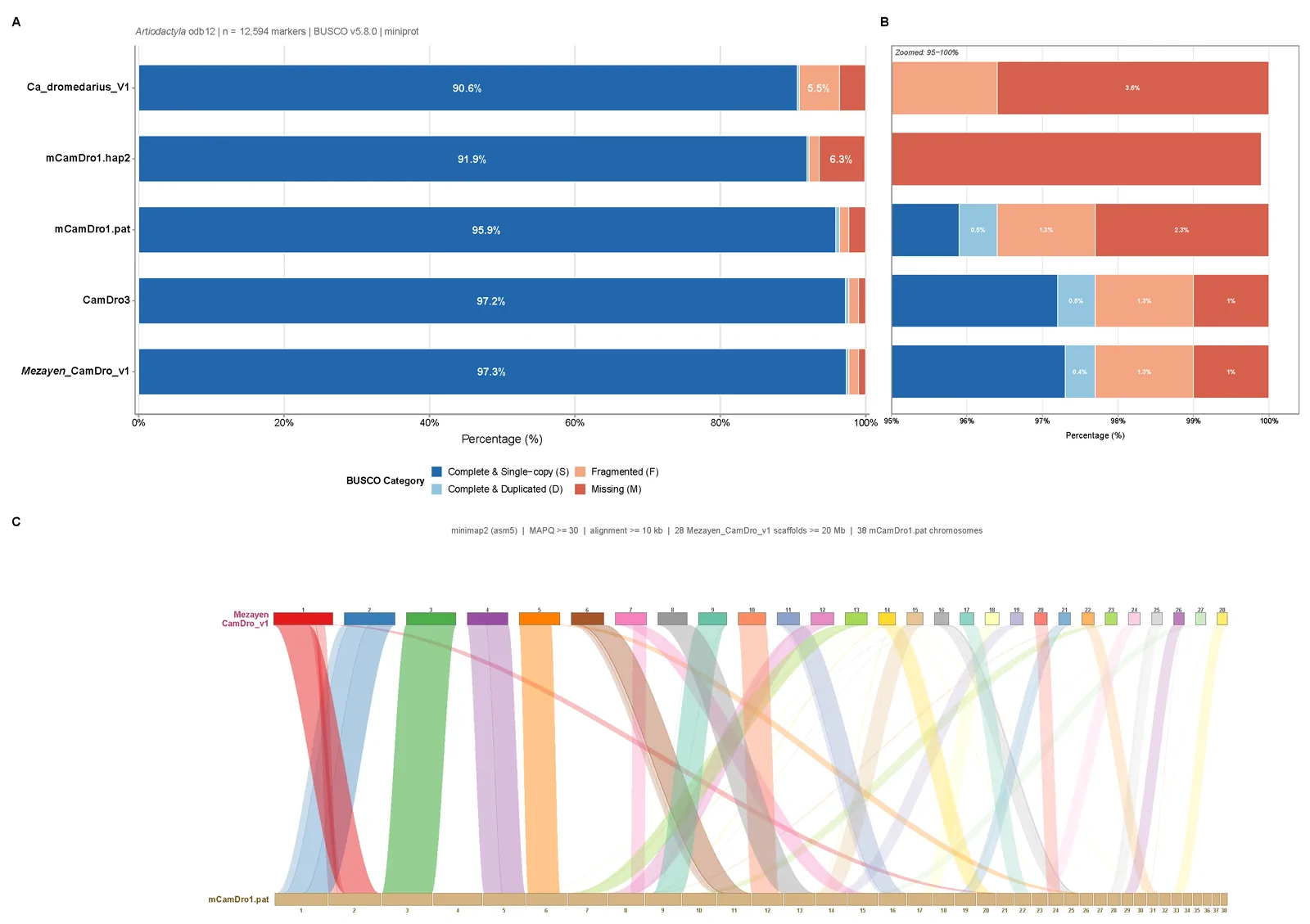
BUSCO assessment and whole-genome synteny of the Mezayen_CamDro_v1 assembly. (**A**) BUSCO completeness scores for five dromedary camel genome assemblies (*Artiodactyla* odb12; OrthoDB v12; 12,594 BUSCOs), ordered from lowest (bottom) to highest (top) by completeness; complete numerical values for all assemblies are provided in Table 1. (**B**) Zoomed view (95–100%) resolving the single-copy, duplicated, fragmented, and missing BUSCO proportions. (**C**) Whole-genome synteny ribbon plot between Mezayen_CamDro_v1 (top; colored by scaffold) and mCamDro1.pat (bottom; tan bars); the 28 largest scaffolds (≥20 Mb, *n* = 28) are shown, with chromosome and scaffold indices ordered by sequence length. Ribbons represent syntenic alignment blocks (minimap2 asm5; alignment ≥ 10 kb; MAPQ ≥ 30). Across the filtered alignments, 98.63% of the assembly aligned to the reference in 390 syntenic blocks (median block length 322 kb) spanning all 38 reference chromosomes; per-chromosome coverage is given in Supplementary Table S2. Chromosomes 19–38 are covered by scaffolds below the 20 Mb display threshold and are therefore represented in Supplementary Table S2 rather than as ribbons.

Assembly completeness was rigorously assessed using BUSCO with the *Artiodactyla* lineage dataset from OrthoDB v12, comprising 12,594 conserved single-copy orthologous genes. This represents a methodological advance over the previous dromedary genome studies, which employed the Cetartiodactyla ortholog set from OrthoDB v10 (13,335 genes), by providing a more taxonomically refined and up-to-date benchmark for camelid genome assessment. The Mezayen_CamDro_v1 assembly achieved a BUSCO completeness score of 97.7%, with 97.3% single-copy complete, 0.4% duplicated, 1.3% fragmented, and only 1.0% missing orthologs (Table 1; Figure 1A,B). These results reflect the efficacy of the hybrid assembly pipeline integrating PacBio long-read assembly, Illumina short-read polishing, and 10x Genomics scaffolding.

To complement the BUSCO assessment, assembly quality was further evaluated using reference-free k-mer and read-mapping approaches. Reference-free k-mer analysis with Merqury yielded a consensus quality value (QV) of 36.63, corresponding to an estimated consensus accuracy of approximately 99.978%, and a k-mer completeness of 95.46%. GenomeScope2 modelling of the Illumina k-mer spectrum estimated a haploid genome size of approximately 2.21 Gb and a heterozygosity of 1.19–1.24%. Read mapping against the final assembly provided strong independent support: Illumina reads showed 99.36% primary mapping and 94.31% proper pairing, with a MAPQ ≥20 weighted mean depth of 59.59×, while PacBio reads showed a 97.77% primary read-mapping rate (21,913,144 of 22,414,076 reads) and a single dominant coverage peak at approximately 80× (mean ∼84×), consistent with a well-supported assembly lacking extensive retained alternate haplotypes. Residual haplotypic duplication was low: although purge_dups initially flagged 3.31 Mb (0.165%) of the assembly as haplotig, independent depth and pairwise-alignment validation supported only two small scaffolds totalling 70,365 bp (0.0035% of the assembly) as strong residual haplotigs. Together, these reference-free, read-based, and redundancy analyses provide assembly-quality support that is independent of BUSCO gene-space completeness.

Illumina polishing with Pilon improved base-level consensus quality while leaving assembly contiguity essentially unchanged. Merqury consensus QV increased from 36.08 before polishing to 36.63 after polishing, assembly-only error k-mers decreased by 11.9% (10,384,667 to 9,147,576), and k-mer completeness increased from 95.37% to 95.46%. A QUAST comparison of the pre- and post-Pilon assemblies showed an unchanged contig count (323), an unchanged L50 (13), and only a marginal change in contig N50 (40,039,755 bp to 40,037,053 bp); the retained Pilon change log recorded 247,311 correction records accounting for a net length change of −274,411 bp. As the initial contigs were generated from PacBio CLR reads, which carry a higher per-base error rate than HiFi reads, this polishing step appropriately corrected residual consensus errors without materially altering assembly structure. The post-Pilon assembly constitutes the polished sequence carried forward to scaffolding and represents the final assembly.

Comparative BUSCO analysis against four publicly available dromedary camel genome assemblies demonstrated that Mezayen_CamDro_v1 is among the most contiguous draft genomic resources currently available for *Camelus dromedarius*. It ties with the North African Dromedary (CamDro3; GCA_000803125.3) [10] for the highest overall completeness (97.7%), while achieving a single-copy BUSCO score (97.3%) comparable to the highest among the assemblies evaluated and the joint-lowest missing gene rate (1.0%). In comparison, the Arabian Dromedary assembly by Wu et al. [4] (Ca_dromedarius_V1; GCA_000767585.1) achieved only 90.9% completeness with 3.6% missing genes, while the haplotype-resolved assemblies from the Vertebrate Genomes Project, mCamDro1.hap2 (GCA_036321565.1) and mCamDro1.pat (GCA_036321535.1), achieved 92.3% and 96.4% completeness with 6.3% and 2.3% missing genes, respectively (Table 1; Figure 1A,B). The substantially lower completeness of Ca_dromedarius_V1 is consistent with its older assembly methodology based solely on short-read Illumina sequencing, which limits contiguity and gene-space recovery. The relatively lower score of mCamDro1.hap2 compared to its paternal counterpart (mCamDro1.pat) likely reflects inherent asymmetry in haplotype-resolved trio binning assemblies. Together, these results establish Mezayen_CamDro_v1 as a high-quality draft genomic resource for the Arabian camel, with gene-content completeness comparable to the currently available dromedary assemblies evaluated.

Genome-wide screening revealed no evidence of substantial contamination or redundancy. Kraken2 identified no confidently non-eukaryotic scaffolds at confidence thresholds of 0.05 or 0.10, and non-eukaryotic assignments in the raw Illumina and PacBio reads were limited to parts-per-million levels. Integration of scaffold GC content, coverage, and taxonomic classification with BlobTools2 flagged no contaminant scaffolds; high-depth outliers were consistent with repetitive or high-copy host sequences rather than foreign DNA. Comparison of an independently assembled mitochondrial genome (16,609 bp) against the nuclear assembly detected no complete or near-complete redundant mitochondrial scaffold; the principal mitochondrial-homologous locus was supported as a nuclear mitochondrial insertion (NUMT) by normal nuclear flanking depth (∼67–69×) and nine spanning PacBio subreads representing six independent molecules. NCBI FCS-adaptor detected no adaptor or vector contamination and recommended no trimming, retaining all 278 sequences and the full assembly length. After independent validation, residual haplotypic duplication was limited to 70,365 bp (0.0035% of the assembly). Per-scaffold results for all analyses are provided in Supplementary Table S1.

To further validate the structural integrity of the Mezayen_CamDro_v1 assembly, whole-genome synteny was assessed against mCamDro1.pat (GCA_036321535.1) as a chromosome-level reference. Pairwise alignments generated using minimap2 revealed broad syntenic conservation between the 28 largest Mezayen_CamDro_v1 scaffolds (≥20 Mb) and the 38 chromosomes of mCamDro1.pat (Figure 1C). The majority of Mezayen_CamDro_v1 scaffolds displayed clear one-to-one correspondences with individual reference chromosomes. A minority of cross-chromosomal alignments were also observed; because such alignments can arise from repetitive or homologous sequences, segmental duplications, ambiguous alignments, or scaffolding artifacts, as well as genuine structural rearrangement, we do not interpret them as specific structural events without independent validation. Across the filtered alignments (≥10 kb, MAPQ ≥ 30), 98.63% of the assembly aligned to the mCamDro1.pat reference in 390 syntenic blocks (median block length 322 kb; mean 5.10 Mb), all as unique primary alignments. Per-chromosome alignment coverage spanned all 38 reference chromosomes at predominantly 85–100% coverage (Supplementary Table S2). The 28 scaffolds ≥ 20 Mb shown in Figure 1C represent the chromosome-scale portion of the assembly (75.8% of total length); smaller scaffolds are omitted from the ribbon plot for clarity but are included in the quantitative summary. The absence of ribbon connections for chromosomes 19–38 in Figure 1C reflects the 20 Mb display threshold rather than the absence of alignment: these chromosomes are covered by smaller scaffolds that fall below the plotting threshold, and all 38 reference chromosomes are represented in the quantitative synteny summary (Supplementary Table S2). This highlights a clear opportunity for future chromosome-level scaffolding using Hi-C or optical mapping technologies.

### 3.2. Genome Annotation

**Figure 2:**
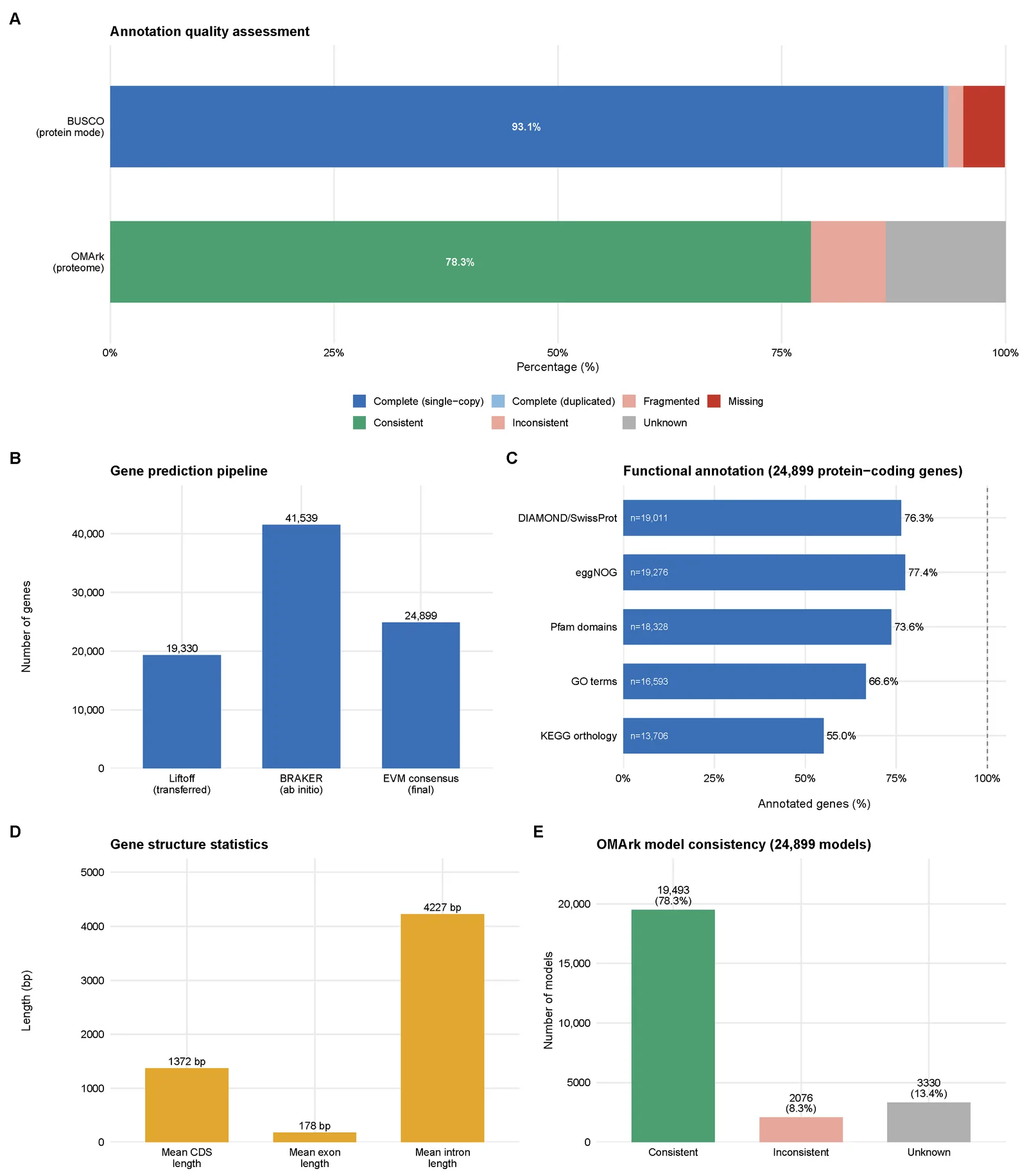
Genome annotation summary of the Mezayen_CamDro_v1 assembly (*Camelus dromedarius*, Majaheem type). (**A**) Annotation quality assessed with BUSCO (protein mode; artiodactyla_odb12) and OMArk (OMA LUCA database). BUSCO colours: complete single-copy (blue), duplicated (light blue), fragmented (salmon), missing (red). OMArk colours: consistent (green), inconsistent (salmon), unknown (grey). (**B**) Protein-coding gene counts at each stage of the annotation pipeline: Liftoff (homology-based transfer), BRAKER (ab initio), and EvidenceModeler (final consensus). (**C**) Functional annotation coverage across five databases: DIAMOND BLASTp/UniProt Swiss-Prot, eggNOG, Pfam, Gene Ontology, and KEGG orthology; dashed line indicates 100% coverage. (**D**) Mean lengths of coding sequences (CDS), exons, and introns in the consensus gene set. (**E**) OMArk model-level consistency classification of the 24,899 predicted proteins.

Gene models were transferred from the conspecific mCamDro1.pat reference (GCF_ 036321535.1) using Liftoff: of 28,257 source genes, 25,840 were mapped and 2417 remained unmapped, including 19,330 mapped protein-coding genes. Independent protein-supported ab initio prediction with BRAKER produced 41,539 raw gene features. Integration of the Liftoff, AUGUSTUS, and GeneMark predictions with EvidenceModeler yielded a consensus set of 24,899 non-redundant protein-coding gene models, comprising 24,899 representative mRNAs, 191,965 CDS, and 191,965 exon features (Figure 2B). Each gene is represented by a single transcript; alternative isoforms, non-coding RNA classes, and pseudogenes are not represented in the final consensus, which is therefore a protein-coding structural annotation.

Gene-structure analysis of the consensus set revealed a mean of 7.71 exons per gene (median 5), with 8083 genes (32.46%) comprising a single exon. The mean coding sequence (CDS) length was 1372 bp, with a mean exon length of 178 bp and a mean intron length of 4227 bp, reflecting the intron-rich gene architecture typical of mammalian genomes (Figure 2D). The total CDS content spanned 34.17 Mb. Predicted proteins ranged from 49 to 34,863 amino acids (median 315 aa; mean 456 aa), and 23,681 models (95.11%) contained both an initiating methionine and a terminal stop codon with no internal stop codons.

To evaluate sensitivity to the EVM weighting scheme, Liftoff:AUGUSTUS:GeneMark weights of 5:2:2, 3:2:2, and 1:1:1 were compared, producing 24,899, 24,580, and 23,343 gene models with protein-mode BUSCO completeness (artiodactyla_odb12) of 93.7%, 77.5%, and 70.9%, respectively; the 5:2:2 scheme was retained. For the selected annotation, protein-mode BUSCO completeness was 93.7% (single-copy 93.1%, duplicated 0.5%), with 1.7% fragmented and 4.6% missing (Figure 2A). Using same-strand reciprocal CDS-base overlap at an 80% threshold, 71.75% of final models were supported by a Liftoff model, 56.60% by a BRAKER model, and 32.51% by both.

Functional annotation of the 24,899 protein-coding genes assigned an annotation in at least one of UniProtKB/Swiss-Prot or eggNOG to 19,451 models (78.12%; Figure 2C). Of these, 18,836 (75.65%) were annotated in both databases; DIAMOND BLASTp against Swiss-Prot assigned descriptions to 19,011 genes (76.35%) and eggNOG-mapper to 19,276 genes (77.42%), including Pfam domains for 18,328 genes (73.61%), GO terms for 16,593 genes (66.64%), and KEGG orthology assignments for 13,706 genes (55.05%). The remaining 5448 models (21.88%) had no detectable homolog in either database; these represent predicted models without database-level functional support rather than validated novel genes; UniProtKB/Swiss-Prot and eggNOG are complementary but not independent sources of evidence, and absence from both does not establish novelty, expression, or lineage specificity. These models may include lineage-specific genes alongside prediction artifacts, gene fragments, or models lacking expression support.

An independent proteome assessment with OMArk (v0.4.1) placed the predicted proteome within the *Artiodactyla* lineage, detected 95.11% of conserved *Artiodactyla* orthologous groups, and identified no likely contaminants. At the whole-proteome level, 19,493 models (78.29%) were classified as consistent with the *Artiodactyla* lineage, 2076 (8.34%) as inconsistent, and 3330 (13.37%) as unknown (Figure 2E); the reduced consistency was concentrated among BRAKER-only models, whereas Liftoff-supported models remained approximately 97–98% consistent. Collectively, these analyses establish the Mezayen_CamDro_v1 annotation as a comprehensive protein-coding resource, while distinguishing the high-confidence, homology-supported core from a lower-confidence, ab initio-derived fraction.

### 3.3. Repeat Element and Simple Sequence Repeat Content

**Figure 3:**
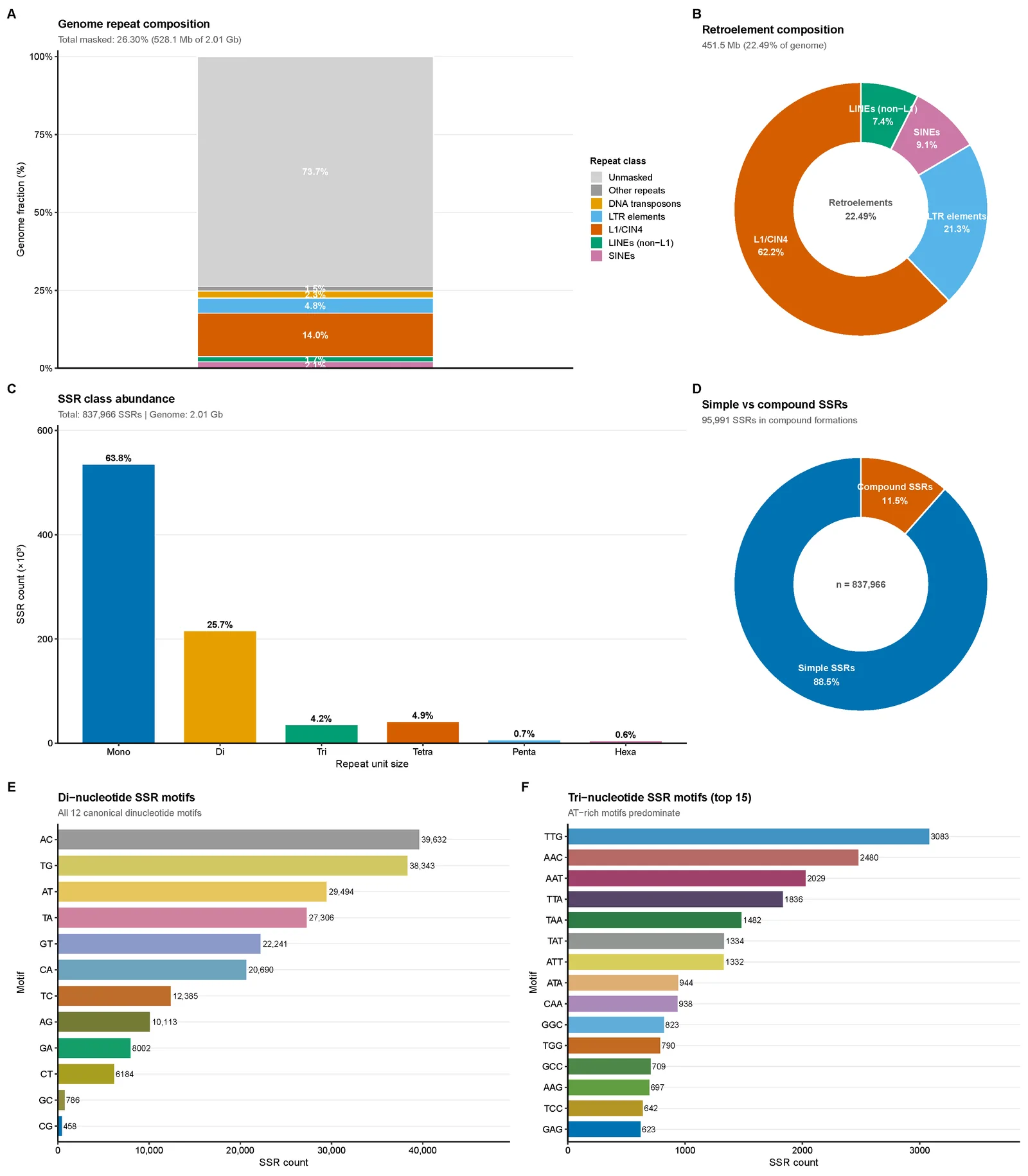
Repeat element and simple sequence repeat (SSR) characterization of the *Mezayen*_ CamDro_v1 genome (*Camelus dromedarius*, Majaheem type). (**A**) Genome-wide repeat composition showing the proportion of each repeat class relative to the total assembly size (2.01 Gb); total masked fraction: 26.30% (528.1 Mb). (**B**) Retroelement sub-class breakdown showing the relative contribution of L1/CIN4, LTR elements, SINEs, and non-L1 LINEs to the total retroelement content (451.5 Mb; 22.49% of the genome). (**C**) Abundance of SSR classes identified by MISA, grouped by repeat unit size (mono- through hexanucleotide); percentages above bars indicate the proportion of each class relative to the total SSR count (837,966 SSRs). (**D**) Proportion of simple versus compound SSRs; compound SSRs are defined as two or more SSR motifs separated by no more than 100 bp. (**E**) Frequency distribution of all 12 canonical dinucleotide SSR motifs, ranked by total count. (**F**) Frequency distribution of the 15 most abundant trinucleotide SSR motifs, illustrating the predominance of AT-rich repeat units. SSR, simple sequence repeat; SINE, short interspersed nuclear element; LINE, long interspersed nuclear element; LTR, long terminal repeat.

Repetitive element annotation of the *Mezayen*_CamDro_v1 assembly revealed that 26.30% of the genome (528.1 Mb of 2.01 Gb) is composed of repetitive sequences (Figure 3A). Retroelements constitute the dominant repeat class, accounting for 22.49% of the total genome (451.5 Mb). Within the retroelement fraction, long interspersed nuclear element (LINE) L1 and CIN4 elements represented the largest sub-class (L1/CIN4; 280.4 Mb; 13.97%), followed by long terminal repeat (LTR) elements (96.0 Mb; 4.78%), short interspersed nuclear elements (SINEs) (41.3 Mb; 2.06%), and non-L1 LINEs (33.5 Mb; 1.67%) (Figure 3B). CIN4 is a Class I LINE retroelement and was grouped with L1-related elements in this study. DNA transposons comprised an additional 2.30% of the genome (46.1 Mb). This repeat content, based on a de novo RepeatModeler library, represents a conservative lower-bound estimate for a scaffold-level assembly; highly diverged or fragmented elements, and any *Camelus*-specific families not fully captured by the custom library, may result in a modest underestimate. A more exhaustive comparative repeat characterization, including cross-comparison with Dfam and Repbase and analysis of transposable-element divergence and relative ages, was beyond the descriptive scope of this resource and represents a direction for future work.

A total of 837,966 SSRs were identified across 272 of the 278 assembled scaffolds, yielding a genome-wide SSR density of 417.4 SSRs/Mb (calculated over the full 2.01 Gb assembly). Mononucleotide repeats were the most abundant SSR class, comprising 63.8% (534,935) of all identified SSRs, followed by dinucleotide repeats (25.7%; 215,634), tetranucleotide repeats (4.9%; 41,446), trinucleotide repeats (4.2%; 35,135), pentanucleotide repeats (0.7%; 6149), and hexanucleotide repeats (0.6%; 4667). Of the total SSRs identified, 741,975 (88.5%) occurred as simple isolated SSRs, while 95,991 (11.5%) were present in compound formations consisting of two or more adjacent SSR motifs separated by no more than 100 bp (Figure 3D).

Analysis of dinucleotide motif composition revealed that AC and TG motifs were the most frequently occurring dinucleotide SSRs (39,632 and 38,343, respectively), followed by AT (29,494), TA (27,306), GT (22,241), and CA (20,690) motifs (Figure 3E). CG and GC dinucleotide motifs were the least abundant, with 458 and 786 occurrences, respectively, consistent with the well-documented CpG suppression observed across vertebrate genomes. Among trinucleotide motifs, AT-rich sequences predominated, with TTG (3083), AAC (2480), and AAT (2029) representing the three most frequent motifs (Figure 3F).

## 4. Discussion

The Arabian camel (*Camelus dromedarius*) occupies a central role in the cultural and economic fabric of the Arabian Peninsula, particularly through the Mezayen beauty contest tradition, which has grown into a major industry spanning Saudi Arabia, Kuwait, and the UAE [6]. Selective breeding for Mezayen competition has intensified demand for camels exhibiting precise morphological standards such as coat color, ear length, neck curvature, and body proportion, traits that are presumed heritable but not yet quantified (e.g., through heritability estimates) and remain poorly characterized at the genomic level [8]. Despite its economic and cultural significance, the Mezayen camel lacked a reference genome from a Mezayen individual prior to this study, representing a critical gap in camelid genomics. Here, we present the first draft scaffold-level genome assembly of a male Mezayen dromedary (Majaheem type), designated Mezayen_CamDro_v1, generated using an integrative multi-platform strategy that combines PacBio long-read sequencing, Illumina short-read polishing, and 10x Genomics linked-read scaffolding.

The Mezayen_CamDro_v1 assembly achieved a total sequence length of 2.01 Gb, a contig N50 of 40.04 Mb, and a BUSCO completeness score of 97.7% against the Artiodactyla OrthoDB v12 dataset, placing it among the most contiguous dromedary assemblies among those evaluated. The genome size and GC content (41.5%) are consistent with previously reported dromedary genome characteristics [4,10]. Notably, Mezayen_CamDro_v1 achieved the highest contig N50 of all five dromedary assemblies evaluated, including the chromosome-level CamDro3 assembly [10] and the haplotype-resolved mCamDro1.pat assembly from the Vertebrate Genomes Project, reflecting the structural contiguity gains afforded by PacBio long-read data. The older Arabian dromedary assembly by Wu et al. [4], generated solely from Illumina short reads, achieved only 90.9% BUSCO completeness and a contig N50 of 0.07 Mb, thereby demonstrating the substantial improvements enabled by hybrid assembly strategies incorporating long-read data [10]. Whole-genome synteny analysis against the chromosome-level mCamDro1.pat reference revealed broad conservation across the 28 largest Mezayen_CamDro_v1 scaffolds, consistent with the high synteny previously reported between dromedary and Bactrian camel genomes, and between camelid and bovine genomes [4].

Genome annotation yielded 24,899 non-redundant protein-coding gene models, within the range reported for other dromedary and Bactrian assemblies [4,10,47,48], with functional annotation assigned to 78.12% of models across UniProt/SwissProt, Gene Ontology, and KEGG databases. A substantial fraction (5448 models; 21.88%) lacked detectable homology in both databases; these represent predicted models without database-level functional support rather than validated novel genes, and their status will require transcriptomic and comparative validation. Repetitive element analysis revealed that 26.30% of the genome comprises repeat sequences, dominated by retroelements, particularly LINE/L1 and CIN4 elements, consistent with the repeat-rich genomic landscapes previously characterized across the genus *Camelus*, where Class I elements have been consistently reported as the predominant component of the repetitive genome fraction [49,50]. The mononucleotide repeats comprise the most abundant class (63.8%), consistent with the dominance of mono- and di-nucleotide motifs previously reported in dromedary camel genomes [51], and provide a catalogue of candidate SSR loci for future polymorphism validation, which, once genotyped in a representative population, could support breed traceability, parentage verification, and marker-assisted selection, as SSR markers have been recognized as a valuable genomic resource for genetic diversity investigation and breeding program development in dromedary camels [50]. We emphasize that the SSR loci and gene models reported here constitute candidate genomic resources; their validation as polymorphic markers and any association with Majaheem phenotypic traits will require genotyping and phenotyping in representative populations before they can inform selection programs. A further limitation is that the annotation relied on homology-based transfer and protein-evidence ab initio prediction without RNA-seq support; the absence of transcriptomic data limits the resolution of isoforms and exon–intron boundaries. Future integration of dromedary RNA-seq, including publicly available multi-tissue and long-read transcriptomic datasets, would refine the gene models, although such data derive from different individuals and tissues and would need to be interpreted with caution.

The genomic resources presented here provides the framework needed to characterize the heritable basis of Majaheem phenotypic traits in future work. Morphometric studies have demonstrated phenotypic distinctions between Majaheem and Malaween camels in torso shape, body size, and coat color [6,8], and a genome-wide study of dromedary camels from Africa and the Arabian Peninsula has identified selection signatures in genes associated with coat color pigmentation, immune response, and metabolic adaptation [11,12], However, the present assembly does not itself resolve these traits; rather, it enables the population-level association and resequencing studies through which the nuclear genomic basis of Majaheem-specific morphological differences could be investigated, which remains unresolved [7]. Mitochondrial diversity analyses have further revealed structured haplogroup variation among Arabian Peninsula dromedary populations [9,52], and the present nuclear genome assembly now provides the complementary framework needed to extend these findings into whole-genome population and association studies. Similar to reference-anchored approaches that have resolved domestication history and migration routes in Bactrian camels [53], the Mezayen_CamDro_v1 assembly provides a reference framework that may facilitate future population-level resequencing, association studies, and selection-scan analyses into the selective breeding history and candidate variants underlying competition traits in dromedary populations across the Gulf region, provided that adequate sample sizes and reliable phenotype records are generated [8,11].

While the Mezayen_CamDro_v1 assembly represents a significant advance in camelid genomics, several aspects of the current work warrant further consideration. The scaffold-level nature of Mezayen_CamDro_v1 limits direct chromosomal comparisons and haplotype phasing; the absence of synteny ribbon connections to chromosomes 19–38 in Figure 1C reflects the 20 Mb plotting threshold rather than the absence of alignment, as all 38 reference chromosomes were covered in the quantitative synteny analysis. Future work should incorporate Hi-C or optical mapping data to achieve chromosome-level scaffolding, as demonstrated for the CamDro3 assembly [10]. As the assembly derives from a single prize-winning Majaheem male, it captures the genomic composition of one individual and may not represent allelic diversity across the Majaheem type; moreover, selection for competition traits and any associated relatedness or inbreeding in elite breeding lines could influence genome-wide diversity and heterozygosity, which population-level sampling would be needed to assess. Additionally, the assembly represents a single male individual of the Majaheem type; population-level resequencing across the full spectrum of Majaheem and other show-camel types and across geographic populations throughout the Arabian Peninsula will be essential to characterize diversity across these types and identify candidate variants underlying competition traits [11,53]. Despite these limitations, Mezayen_CamDro_v1 constitutes a foundational genomic resource that bridges the rich phenotypic tradition of Mezayen camel breeding and modern genomic-assisted selection programs, with broad applications in conservation, breed authentication, and the study of culturally and economically important traits in these camels.

## 5. Conclusions

This study presents the first draft scaffold-level genome assembly of a Mezayen dromedary camel (*Camelus dromedarius*, Majaheem type), designated Mezayen_CamDro_v1. By integrating PacBio long-read sequencing, Illumina short-read polishing, and 10x Genomics linked-read scaffolding, we produced a 2.01 Gb assembly with a contig N50 of 40.04 Mb and a BUSCO completeness score of 97.7%, the highest contig N50 among the five dromedary assemblies evaluated in this study, despite its scaffold-level rather than chromosome-level assembly status. Genome annotation yielded 24,899 non-redundant protein-coding genes with high functional coverage across UniProt/SwissProt, Gene Ontology, and KEGG databases, alongside a comprehensive characterization of repetitive elements and simple sequence repeats that provides a catalogue of candidate SSR loci for future polymorphism validation and potential use in breed traceability and marker-assisted selection.

The Mezayen_CamDro_v1 assembly establishes a foundational genomic resource for the Majaheem type, which holds considerable cultural and economic importance in the Arabian Peninsula, yet has until now lacked a nuclear genomic reference from a Mezayen individual. Future work incorporating Hi-C or optical mapping data will be required to elevate the assembly to chromosome scale, and integration of RNA-seq evidence, which was not available for the present annotation, will further refine the gene models. Population-level resequencing across the full spectrum of Majaheem and other show-camel types and geographic subpopulations will be essential to identify the candidate variants underlying their defining morphological and pigmentation traits, including loci such as *MC1R* and *ASIP*. Together, these advances will accelerate genomic-assisted selection programs and support the long-term conservation of these esteemed camels.

## Figures and Tables

**Table 1:** General assembly statistics and BUSCO scores for dromedary camel genome assemblies.

Metric	North African Dromedary. Elbers et al. [10]	Arabian Dromedary. Wu et al. [4]	Arabian Dromedary (Maternal Haplotype)	Arabian Dromedary (Paternal Haplotype)	Mezayen Dromedary (Majaheem Type), This Study
Assembly Information					
Accession number	GCA_000803125.3	GCA_000767585.1	GCA_036321565.1	GCA_036321535.1	GCA_037784275.1
Assembly name	CamDro3	Ca_dromedarius_V1	mCamDro1.hap2	mCamDro1.pat	Mezayen_CamDro_ v1
Assembly level	Chromosome	Scaffold	Chromosome	Chromosome	Scaffold
Sex	Female	Male	Male	Male	Male
Geographical origin	North Africa (African breed)	Saudi Arabia (Riyadh)	Qatar	Qatar	Saudi Arabia (Wadi Aldwaser)
Sequencing technology	Illumina HiSeq; Dovetail Hi-C & Chicago; PacBio Sequel	Illumina HiSeq2000	PacBio Sequel II HiFi; Dovetail OmniC	PacBio Sequel II HiFi; Dovetail OmniC	PacBio Sequel; Illumina NextSeq 500; 10x Genomics
Assembly Metrics					
Total sequence length (Gb)	2.17	2.00	2.09	2.31	2.01
Total ungapped length (Gb)	2.15	1.98	2.09	2.31	2.01
Number of contigs	53,084	32,572	383	366	323
Contig N50 (Mb)	0.24	0.07	28.90	31.22	40.04
Contig L50	2637	7850	21	22	13
Number of scaffolds	21,069	32,572	173	171	278
Scaffold N50 (Mb)	70.37	4.19	70.33	72.99	53.40
Scaffold L50	11	132	12	12	12
Number of chromosomes	37	—	36	38	—
GC content (%)	41.5	41.5	41.5	41.5	41.5
BUSCO Assessment (Artiodactyla, OrthoDB v12, 12,594 genes)					
Complete (%)	**97.7**	90.9	92.3	96.4	**97.7**
Single-copy (%)	97.2	90.6	91.9	95.9	**97.3**
Duplicated (%)	0.5	0.3	0.3	0.5	0.4
Fragmented (%)	1.3	5.5	1.4	1.3	1.3
Missing (%)	**1.0**	3.6	6.3	2.3	**1.0**

Abbreviations: Gb, gigabases; Mb, megabases; BUSCO, Benchmarking Universal Single-Copy Orthologs. All assemblies were downloaded from NCBI on 5 April 2026 using the datasets command-line tool v14. BUSCO v5.8.0 was run using the artiodactyla_odb12 lineage dataset (OrthoDB v12, created 1 July 2025, 12,594 genes, gene predictor: miniprot). GCA and GCF denote the GenBank and RefSeq records of the same assembly; the paternal haplotype (mCamDro1.pat) used for gene transfer corresponds to RefSeq accession GCF_036321535.1 (Annotation Release 102). The Mezayen_CamDro_v1 assembly contains 4500 ambiguous (N) bases in 45 fixed 100-bp gaps introduced during 10x scaffolding; its total and ungapped lengths are 2,007,644,109 bp and 2,007,639,609 bp, respectively (identical at the Gb precision shown in the table). Bold values indicate the best-performing assembly for each metric. “—” indicates the metric is not applicable or not reported for scaffold-level assemblies.

## Data Availability

The raw genome sequencing reads generated in this study have been deposited in the NCBI Sequence Read Archive (SRA) under BioProject accession PRJNA1077647. The draft genome assembly of the male Mezayen dromedary camel is available in GenBank under accession GCA_037784275.1 (https://www.ncbi.nlm.nih.gov/datasets/genome/GCA_037784275.1/ (accessed on 10 September 2026)). The protein-coding genome annotation described in this study (GFF3, protein, and CDS files) is available in Figshare at https://doi.org/10.6084/m9.figshare.33193326.v2 (accessed on 10 September 2026). The computational workflow, command-line parameters, configuration files, and software-version information are available in a public GitHub repository at https://github.com/MManee/mezayen-camel-genome (accessed on 10 September 2026).

## Supplementary Materials

The following supporting information can be downloaded at: https://www.mdpi.com/article/10.3390/ani16182905/s1. Table S1: Contamination and redundancy screening of the Mezayen_CamDro_v1 genome assembly; Table S2: Per-chromosome whole-genome synteny coverage of the Mezayen_CamDro_v1 assembly against the mCamDro1.pat reference; Table S3: Sequencing data yield, read metrics, and coverage for the Mezayen_CamDro_v1 genome assembly.
