# Supplementary material for: Draft Genome Assembly and Annotation of a Male Mezayen Dromedary Camel of the Majaheem Type (Camelus dromedarius)
Source: Animals (Basel). 2026 Sep 15;16(18):2905. doi: 10.3390/ani16182905 (PMC13603154; doi:10.3390/ani16182905)
Supplement: Supplementary file 1 [file animals-16-02905-s001.zip › Supplementary_Table_S1.pdf]

Supplementary Table S1. Contamination and redundancy screening of the Mezayen_CamDro_v1 genome assembly (Camelus dromedarius, Majaheem type).

Screening was performed at the scaffold level using complementary approaches. No evidence of substantial non-eukaryotic contamination, actionable adaptor/vector contamination, redundant mitochondrial sequence, or extensive haplotypic duplication was detected. Panel (a) summarizes each analysis and its genome-wide result; panel (b) lists the individual scaffolds flagged for follow-up (coverage outliers, the principal mitochondrial-homologous locus, and validated residual haplotigs). The complete per-scaffold table (all 278 scaffolds; scaffold ID, length, GC, MAPQ≥20 depth, fold-over-nuclear-median, Kraken2 status/assignment, mtDNA hit, and purge_dups class) is provided as the accompanying data file.

(a) Summary of analyses and genome-wide results

Analysis	Tool (version)	Result	Assessment
Consensus accuracy (QV)	Merqury v1.3	QV 36.63; estimated consensus accuracy ~99.978%	Strong (draft)
k-mer completeness	Merqury v1.3	95.46% of reliable read k-mers represented	High
Heterozygosity / genome size	GenomeScope2 v2.1.0	Heterozygosity 1.19–1.24%; haploid size ~2.21 Gb	Informative
Illumina read mapping	BWA-MEM2 v2.2.1	99.36% primary mapped; 94.31% proper pairs; MAPQ≥20 mean depth 59.59×	Excellent
PacBio read mapping	minimap2 v2.31	97.77% primary read mapping (21,913,144/22,414,076); dominant depth peak ~80×	Excellent
Taxonomic screen (assembly)	Kraken2 v2.17.1 (PlusPF)	No confidently bacterial/fungal/archaeal/viral scaffolds at confidence 0.05 or 0.10	Reassuring
Taxonomic screen (raw reads)	Kraken2 v2.17.1 (PlusPF)	Non-eukaryotic assignments only at ppm level (Illumina ~17–20 ppm; PacBio ~1.7 ppm)	Reassuring
GC + coverage integration	BlobTools2 v4.5.1	Assembly GC 41.45%; no scaffold flagged by combined GC/coverage/taxonomy	Clean
Mitochondrial redundancy	BLASTN; NOVOPlasty (mtDNA 16,609 bp)	No complete/near-complete redundant mt scaffold; principal locus supported as NUMT	Strong
Haplotypic duplication	purge_dups v1.2.6	3.31 Mb (0.165%) initially flagged; 70,365 bp (0.0035%) strongly supported after validation	No extensive duplication
Adaptor/vector screen	NCBI FCS-adaptor v0.5.5	No adaptor/vector calls; no trimming; all 278 sequences / 2,007,644,109 bp retained	Clean

(b) Individual scaffolds flagged for follow-up

High-depth coverage outliers (top five; MAPQ≥20 depth vs. nuclear median 57.39×). None was confidently non-eukaryotic by Kraken2; high depth is consistent with repetitive/high-copy host sequence.

Scaffold	Length (bp)	MAPQ≥20 depth	GC (%)	Fold over median
JBAJJD010000245.1	31,762	1,787.12×	38.77	31.14×
JBAJJD010000277.1	248,922	1,580.94×	42.94	27.55×
JBAJJD010000125.1	53,265	1,531.01×	45.19	26.68×
JBAJJD010000278.1	119,801	1,225.39×	56.18	21.35×
JBAJJD010000029.1	839,285	974.45×	39.12	16.98×

Validated residual haplotigs (of 15 whole-scaffold purge_dups candidates, only these two showed the combined high-identity / low-depth pattern expected for strong residual haplotigs; total 70,365 bp = 0.0035% of the assembly).

Candidate scaffold	Matched scaffold	Aligned fraction	Best identity	PacBio depth ratio
JBAJJD010000117.1	JBAJJD010000084.1	100.00%	99.03%	0.075
JBAJJD010000166.1	JBAJJD010000172.1	90.80%	99.44%	0.227

Principal mitochondrial-homologous locus (on 76.5-Mb nuclear scaffold JBAJJD010000089.1). Normal nuclear flanking depth with locally inflated mt-like depth from short-read cross-mapping; nine spanning PacBio subreads (six independent ZMWs) support an integrated NUMT rather than mitochondrial contamination.

Region on JBAJJD010000089.1	Approx. Illumina depth
Left nuclear flank (16,153,000–16,163,000)	67.28×
Near-identical mt-like segment (16,163,782–16,168,883)	5,619.65×
Diverged mt-like segment (16,168,819–16,177,720)	3,888.79×
Right nuclear flank (16,179,000–16,189,000)	68.83×

Abbreviations: QV, consensus quality value; GC, guanine–cytosine content; MAPQ, mapping quality; ppm, parts per million; NUMT, nuclear mitochondrial insertion; ZMW, zero-mode waveguide. Nuclear depth baseline = median MAPQ≥20 depth among scaffolds ≥10 Mb (57.39×). All analyses were performed on the final 278-scaffold assembly (GCA_037784275.1; 2,007,644,109 bp).
