# Supplementary material for: Draft Genome Assembly and Annotation of a Male Mezayen Dromedary Camel of the Majaheem Type (Camelus dromedarius)
Source: Animals (Basel). 2026 Sep 15;16(18):2905. doi: 10.3390/ani16182905 (PMC13603154; doi:10.3390/ani16182905)
Supplement: Supplementary file 1 [file animals-16-02905-s001.zip › Supplementary_Table_S2.pdf]

Supplementary Table S2. Per-chromosome whole-genome synteny coverage of the Mezayen_CamDro_v1 assembly against the mCamDro1.pat reference (GCA_036321535.1).

Pairwise alignments were generated with minimap2 (-x asm5) and filtered to a minimum alignment length of 10 kb and mapping quality ≥30. Coverage is the proportion of each reference chromosome spanned by the union of filtered alignment blocks. All 38 reference chromosomes are represented; chromosomes are numbered by descending length. Overall, 98.63% of the assembly aligned to the reference across 390 syntenic blocks (median block length 322 kb; mean 5.10 Mb), all as unique primary alignments.

Chr.	Reference sequence	Length (Mb)	Coverage (%)	Blocks
1	CM070310.1	123.3	96.6	3
2	CM070311.1	122.7	98.0	8
3	CM070312.1	119.2	97.8	1
4	CM070346.1	114.2	89.6	110
5	CM070313.1	98.5	97.2	4
6	CM070314.1	94.6	99.3	5
7	CM070315.1	92.1	84.5	9
8	CM070316.1	84.8	99.4	6
9	CM070317.1	83.8	93.3	5
10	CM070318.1	80.5	92.2	7
11	CM070319.1	79.0	98.9	5
12	CM070320.1	73.0	87.7	6
13	CM070321.1	72.9	99.2	4
14	CM070322.1	71.1	95.5	6
15	CM070323.1	70.5	95.1	6
16	CM070324.1	63.8	86.7	6
17	CM070325.1	58.4	89.7	6
18	CM070326.1	48.8	100.0	2
19	CM070327.1	48.0	85.3	6
20	CM070328.1	43.2	90.6	4
21	CM070329.1	39.8	95.6	3
22	CM070330.1	38.2	87.7	5
23	CM070331.1	35.8	76.4	7
24	CM070332.1	34.6	84.5	6
25	CM070333.1	33.1	97.7	3
26	CM070334.1	31.0	70.6	6
27	CM070335.1	29.2	93.1	5
28	CM070336.1	29.1	90.7	3
29	CM070337.1	27.9	89.6	4
30	CM070338.1	27.7	98.5	2
31	CM070339.1	25.4	98.9	2
32	CM070340.1	25.2	96.2	3
33	CM070341.1	23.9	99.5	3
34	CM070347.1	20.6	56.2	5
35	CM070342.1	20.4	78.7	5
36	CM070343.1	20.1	99.9	6
37	CM070344.1	17.1	35.1	7
38	CM070345.1	14.4	99.5	9
**Total**	**38 chromosomes**	**2135.9**	**92.6**	**375**

Notes: Chr., chromosome index (by descending reference length); Blocks, number of filtered syntenic alignment blocks mapping to that chromosome. The "Total" coverage (92.6%) is the length-weighted mean across reference chromosomes; the assembly-side figure (98.63% of the 2.008-Gb assembly aligned) is reported in the main text. Nine additional unplaced reference scaffolds received minor alignments (<7% coverage each) and are not listed. Alignments were generated with minimap2 v2.30 and processed as described in Section 2.3.
