# Supplementary material for: Draft Genome Assembly and Annotation of a Male Mezayen Dromedary Camel of the Majaheem Type (Camelus dromedarius)
Source: Animals (Basel). 2026 Sep 15;16(18):2905. doi: 10.3390/ani16182905 (PMC13603154; doi:10.3390/ani16182905)
Supplement: Supplementary file 1 [file animals-16-02905-s001.zip › Supplementary_Table_S3.pdf]

Supplementary Table S3. Sequencing data yield, read metrics, and coverage for the Mezayen_CamDro_v1 genome assembly (Camelus dromedarius, Majaheem type).

Values were verified from the retained raw FASTQ files and archived assembly, polishing, scaffolding, and read-mapping records. Theoretical input coverage is reported relative to the 3-Gb genome-size parameter used in the NextDenovo assembly configuration; mapping-based depth is reported relative to the final 2.008-Gb assembly. The two PacBio CLR datasets (pacbio_1 and pacbio_2) together total 384.54 Gb; the initial de novo assembly used pacbio_1 only. Coverage values are labelled by type and denominator and should not be compared across different denominators.

(a) Raw sequencing yield and read metrics

Platform / dataset	Reads	Raw bases (Gb)	Mean length	Read N50 / notes
Illumina (4 lanes, PE)	1,003,845,298	151.58	151 bp	fixed-length 151 bp
PacBio CLR, pacbio_1	22,414,076	226.46	10.10 kb	N50 13.39 kb (assembly input)
PacBio CLR, pacbio_2	11,514,636	158.08	13.73 kb	N50 19.98 kb (not used for assembly)
PacBio CLR, total	33,928,712	384.54	—	pacbio_1 + pacbio_2
10x Genomics	736,741,550	111.25	151 bp	fixed-length 151 bp

(b) PacBio assembly-input filtering (pacbio_1, NextDenovo read_cutoff = 1 kb)

Metric	Raw pacbio_1	≥1 kb retained	Removed	Retained %
Reads	22,414,076	21,751,541	662,535	97.04%
Bases (bp)	226,459,268,481	226,119,206,574	340,061,907	99.85%
Mean length (bp)	10,103	10,396	—	—
Read N50 (bp)	13,390	13,411	—	—

Retained ≥1-kb read-length distribution: minimum 1,000 bp; Q1 5,022 bp; median 7,435 bp; Q3 12,348 bp; maximum 310,209 bp.

(c) Coverage and mapping depth by stage

Stage / metric	Value	Basis / notes
PacBio assembly input (theoretical)	75.37×	pacbio_1 ≥1 kb (226.12 Gb) / 3 Gb parameter
PacBio mapping to final assembly	97.77%	21,913,144 / 22,414,076 reads mapped (minimap2 map-pb)
PacBio mapped depth	~84× (peak ~80×)	purge_dups coverage profile on final assembly
PacBio read-to-assembly divergence	4.43%	minimap2 dv proxy (not an instrument error rate)
Illumina final-assembly mapping	99.36%	primary mapped; 94.31% properly paired
Illumina mapped depth	~59.59×	MAPQ≥20 weighted mean, final assembly
Illumina polishing depth (effective)	~16.49×	retained Pilon BAM (lane 1); 33.12 Gb CIGAR-mapped / 2.008 Gb pre-polish
10x scaffolding input (theoretical)	37.08×	111.25 Gb / 3 Gb parameter (55.41× vs final assembly)

Abbreviations: PE, paired-end; CLR, PacBio continuous long reads; N50, length-weighted median read length; MAPQ, mapping quality; dv, minimap2 per-base sequence divergence. Theoretical input coverage uses the 3-Gb NextDenovo genome-size parameter as the denominator; mapping-based depths use the final 2,007,644,109-bp assembly. The minimap2 dv value is reported as a read-to-assembly divergence proxy, as it also reflects heterozygosity and true variants and is not a direct measure of instrument error. No separate read-trimming or quality-filtering step was recovered from the retained workflow records other than the NextDenovo 1-kb PacBio cutoff.
